# 1-Hexyl-1,3,6,8-tetra­aza­tricyclo­[4.3.1.1^3,8^]undecan-1-ium iodide

**DOI:** 10.1107/S1600536811050781

**Published:** 2011-12-03

**Authors:** Augusto Rivera, John Sadat-Bernal, Jaime Ríos-Motta, Karla Fejfarová, Michal Dušek

**Affiliations:** a Departamento de Química, Universidad Nacional de Colombia, Ciudad Universitaria, Bogotá, Colombia; b Institute of Physics ASCR, v.v.i., Na Slovance 2, 182 21 Praha 8, Czech Republic

## Abstract

In the title compound, C_13_H_27_N_4_^+^·I^−^, the ethyl­ene bridge is distorted from the ideal *D*_2*d*_ symmetry wherein an N—C—C—N planar bridge, around whose C—C bond the C—N and C—H bonds are exactly eclipsed, is disordered over two sites with equal occupancies. In both disorder components, the hexyl chain adopts an ideal all-*trans* conformation. In the crystal, adjacent ions are connected by C—H⋯I hydrogen bonds, forming ionic pairs that are further linked into chains along [101] *via* a second C—H⋯I inter­action.

## Related literature

For related structures, see: Rivera *et al.* (2011*a* ▶,*b* ▶). For the preparation of the title compound, see: Rivera *et al.* (2011*b* ▶). For synthetic applications of quaternary ammonium salts, see: Starks (1971 ▶). For bond-length data, see: Allen *et al.* (1987 ▶).
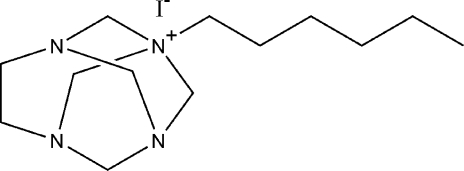


## Experimental

### 

#### Crystal data

C_13_H_27_N_4_^+^·I^−^
*M* *_r_* = 366.3
Monoclinic, 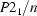

*a* = 8.4914 (4) Å
*b* = 16.1497 (6) Å
*c* = 11.8673 (6) Å
β = 102.690 (5)°
*V* = 1587.65 (13) Å^3^
*Z* = 4
Mo *K*α radiation
μ = 2.01 mm^−1^
*T* = 120 K
0.21 × 0.19 × 0.11 mm

#### Data collection

Agilent Xcalibur Atlas Gemini ultra diffractometer
Absorption correction: multi-scan (*CrysAlis PRO*; Agilent, 2010 ▶), *T*_min_ = 0.930, *T*_max_ = 1.000
6803 measured reflections
6795 independent reflections
4959 reflections with *I* > 3σ(*I*)
*R*_int_ = 0.028

#### Refinement

*R*[*F* ^2^ > 2σ(*F* ^2^)] = 0.034
*wR*(*F* ^2^) = 0.086
*S* = 1.23
6795 reflections
160 parameters
6 restraints
H-atom parameters constrained
Δρ_max_ = 0.71 e Å^−3^
Δρ_min_ = −0.54 e Å^−3^

### 

Data collection: *CrysAlis PRO* (Agilent, 2010 ▶); cell refinement: *CrysAlis PRO*; data reduction: *CrysAlis PRO*; program(s) used to solve structure: *SIR2002* (Burla *et al.*, 2003 ▶); program(s) used to refine structure: *JANA2006* (Petříček *et al.*, 2006 ▶); molecular graphics: *DIAMOND* (Brandenburg & Putz, 2005 ▶); software used to prepare material for publication: *JANA2006*.

## Supplementary Material

Crystal structure: contains datablock(s) global, I. DOI: 10.1107/S1600536811050781/bx2384sup1.cif

Structure factors: contains datablock(s) I. DOI: 10.1107/S1600536811050781/bx2384Isup2.hkl

Supplementary material file. DOI: 10.1107/S1600536811050781/bx2384Isup3.cml

Additional supplementary materials:  crystallographic information; 3D view; checkCIF report

## Figures and Tables

**Table 1:** Hydrogen-bond geometry (Å, °)

*D*—H⋯*A*	*D*—H	H⋯*A*	*D*⋯*A*	*D*—H⋯*A*
C1—H1*a*⋯I1^i^	0.96	2.98	3.913 (3)	164
C3—H3*b*⋯I1^ii^	0.96	3.04	3.925 (2)	154

Symmetry codes: (i) 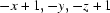; (ii) 
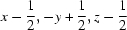
.

**Fig. 1.**
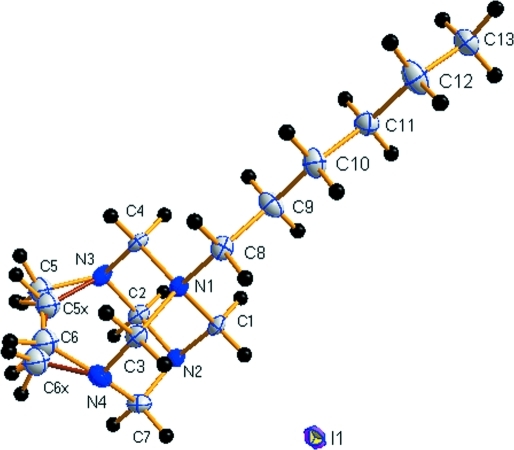
A view of (I) with the numbering scheme. Displacement ellipsoids are drawn at the 30% probability level. The H atoms are shown as small spheres of arbitrary radii.

**Fig. 2.**
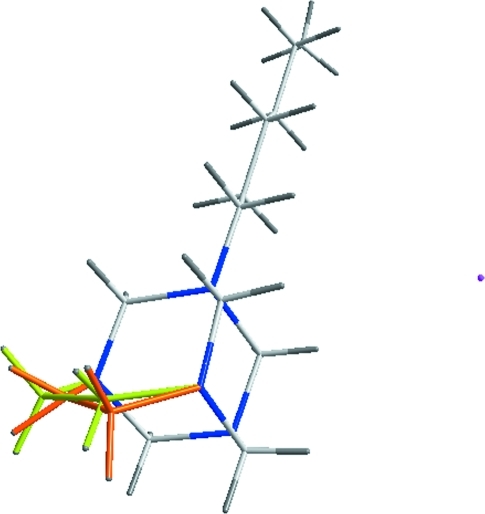
Overlay diagram showing the conformational disorder of the ethylene bridge.

**Fig. 3.**
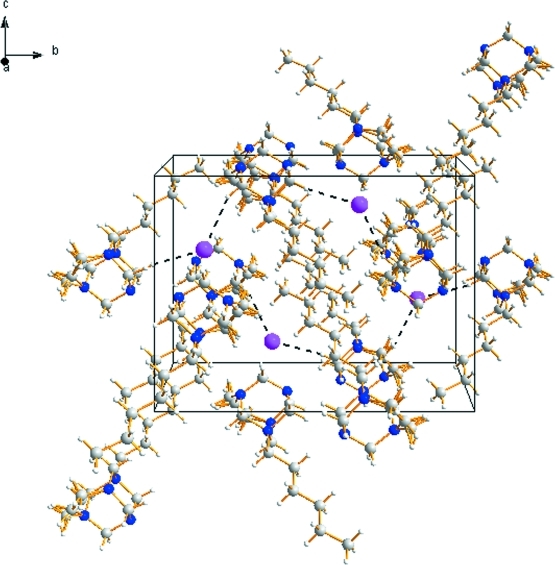
Packing of the ions of the title compound view along a axis. C—H···I hydrogen bonds are drawn as dashed lines.

## supplementary crystallographic information

### Comment

In previous paper we described the synthesis of a series of new *N*-alkylated quaternary ammonium salts derived from the cyclic aminal 1,3,6,8-tetraazatricyclo[4.3.1.1^3,8^]undecane by alkylation with alkyl halides according the Menschutkin reaction (Rivera *et al.*, 2011*b*).

As a part of our interest in complementing the structural information on these quaternary ammonium salts herein we report the results of the X-ray structure determination of the title compound (I). A perspective view of the molecule of the title compound, showing the atomic numbering scheme, is given in Fig. 1. The bridge is distorted from the ideal *D_2 d_* symmetry, and is disordered over two sites (N3—C5—C6—N4 and N3—C5x—C6x—N4) with equal occupancies (Fig. 2). Whereas the N—C—C—N fragment in the first conformer is nearly planar [torsion angle = 0.4 (9)°], the second conformer is slightly twisted out with a N3—C5x—C6x—N4 torsion angle of 9.6 (10)°. In both disorder components the hexyl chain adopts an ideal *all*-*trans* conformation. Bond lengths (Allen *et al.*, 1987) and angles are normal and comparable to the related structure (Rivera *et al.*, 2011*a*). However, the observed C—C bond lengths [C5—C6, 1.439 (10) Å; C5x—C6x, 1.435 (10) Å] are shorter in relation to the mentioned related structure [C—C, 1.475 (4) Å] (Rivera, *et al.* 2011*b*). Moreover, the C—C bonds in the chain tend to be slightly shorter than the average values observed in related structure by 0.015 Å. The most obvious differences with the related structures is the observed disorder of the ethylene fragment in the title compound. This disorder is not observed in related structure (Rivera *et al.*, 2011*a*).

In the crystal, adjacent ions are connected by intermolecular C—H···I hydrogen bonds [C1···I1, 3.913 (3) Å] forming ionic pairs that are further linked into chains along [101] *via* a second intermolecular C—H···I interactions [C3···I3, 3.925 (2) Å] (Table 1, Fig. 3).

### Experimental

The title compound was synthesized according to the published procedure (Rivera *et al.*, 2011*b*). The crystallization was carried out at room temperature by slow evaporation of title compound solution in ethanol.

### Refinement

All hydrogen atoms were added to calculated positions with C–H distance 0.96 Å and refined as riding on their parent atoms. The isotropic atomic displacement parameters of hydrogen atoms were evaluated as 1.2×*U*_eq_ of the parent atom.

Refinement of atomic positions in disordered part was unreliable, probably due to partial overlaps of reflections caused by twinning. No untwinned sample could be found. Therefore, the coordinates of disordered atoms were refined with restrictions on C—C and C—N bond lenghts of 1.46 Å with σ 0.005. During the refinement it was also necessary to fix occupancy of the disordered parts.

### Crystal data

**Table:**

C_13_H_27_N_4_^+^·I^−^	*F*(000) = 744
*M**_r_* = 366.3	*D*_x_ = 1.532 Mg m^−^^3^
Monoclinic, *P*2_1_/*n*	Mo *K*α radiation, λ = 0.71069 Å
Hall symbol: -P 2yn	Cell parameters from 6021 reflections
*a* = 8.4914 (4) Å	θ = 3.0–28.6°
*b* = 16.1497 (6) Å	µ = 2.01 mm^−^^1^
*c* = 11.8673 (6) Å	*T* = 120 K
β = 102.690 (5)°	Prism, colourless
*V* = 1587.65 (13) Å^3^	0.21 × 0.19 × 0.11 mm
*Z* = 4	

### Data collection

**Table:**

Agilent Xcalibur Atlas Gemini ultra diffractometer	6795 independent reflections
Radiation source: Enhance (Mo) X-ray Source	4959 reflections with *I* > 3σ(*I*)
graphite	*R*_int_ = 0.028
Detector resolution: 10.3784 pixels mm^-1^	θ_max_ = 28.7°, θ_min_ = 2.8°
Rotation method data acquisition using ω scans	*h* = −11→11
Absorption correction: multi-scan (*CrysAlis PRO*; Agilent, 2010),	*k* = −21→21
*T*_min_ = 0.930, *T*_max_ = 1.000	*l* = −15→15
6803 measured reflections	

### Refinement

**Table:**

Refinement on *F*^2^	126 constraints
*R*[*F*^2^ > 2σ(*F*^2^)] = 0.034	H-atom parameters constrained
*wR*(*F*^2^) = 0.086	Weighting scheme based on measured s.u.'s *w* = 1/[σ^2^(*I*) + 0.0016*I*^2^]
*S* = 1.23	(Δ/σ)_max_ = 0.010
6795 reflections	Δρ_max_ = 0.71 e Å^−^^3^
160 parameters	Δρ_min_ = −0.54 e Å^−^^3^
6 restraints	

### Special details

**Table:**

Experimental. (CrysAlis PRO; Agilent, 2010), Empirical absorption correction using spherical harmonics, implemented in SCALE3 ABSPACK scaling algorithm.
Refinement. The refinement was carried out against all reflections. The conventional *R*-factor is always based on *F*. The goodness of fit as well as the weighted *R*-factor are based on *F* and *F*^2^ for refinement carried out on *F* and *F*^2^, respectively. The threshold expression is used only for calculating *R*-factors *etc*. and it is not relevant to the choice of reflections for refinement.The program used for refinement, Jana2006, uses the weighting scheme based on the experimental expectations, see _refine_ls_weighting_details, that does not force *S* to be one. Therefore the values of *S* are usually larger than the ones from the *SHELX* program.

### Fractional atomic coordinates and isotropic or equivalent isotropic displacement parameters (Å^2^)

**Table:**

	*x*	*y*	*z*	*U*_iso_*/*U*_eq_	Occ. (<1)
I1	0.76438 (2)	0.145638 (12)	0.653063 (18)	0.03486 (7)	
N1	0.3333 (2)	0.14856 (13)	0.3771 (2)	0.0243 (7)	
N2	0.1997 (3)	0.08915 (14)	0.5186 (2)	0.0288 (8)	
N3	0.0378 (3)	0.14480 (14)	0.3388 (2)	0.0315 (8)	
N4	0.2240 (3)	0.23902 (15)	0.5091 (2)	0.0388 (9)	
C1	0.3335 (3)	0.07917 (16)	0.4647 (2)	0.0271 (9)	
C2	0.0478 (3)	0.08342 (17)	0.4302 (2)	0.0304 (9)	
C3	0.3437 (3)	0.23048 (16)	0.4446 (2)	0.0305 (10)	
C4	0.1724 (3)	0.14102 (17)	0.2875 (2)	0.0264 (8)	
C5	−0.0374 (8)	0.2221 (3)	0.3446 (7)	0.0496 (12)*	0.5
C5x	0.0024 (9)	0.2269 (3)	0.3739 (7)	0.0496 (12)*	0.5
C6	0.0726 (7)	0.2638 (5)	0.4366 (6)	0.0572 (13)*	0.5
C6x	0.0779 (8)	0.2814 (5)	0.4655 (7)	0.0572 (13)*	0.5
C7	0.2155 (4)	0.16748 (18)	0.5817 (3)	0.0362 (11)	
C8	0.4742 (3)	0.14364 (17)	0.3199 (3)	0.0311 (9)	
C9	0.4960 (4)	0.0633 (2)	0.2612 (3)	0.0442 (12)	
C10	0.6358 (4)	0.0646 (2)	0.2041 (3)	0.0449 (12)	
C11	0.6715 (4)	−0.0158 (2)	0.1516 (3)	0.0431 (12)	
C12	0.8106 (4)	−0.0173 (2)	0.0927 (3)	0.0597 (15)	
C13	0.8456 (4)	−0.0991 (2)	0.0453 (3)	0.0559 (15)	
H1a	0.325427	0.026624	0.426054	0.0325*	
H1b	0.432326	0.080962	0.522431	0.0325*	
H2a	0.037822	0.028919	0.397084	0.0365*	
H2b	−0.042195	0.090043	0.46622	0.0365*	
H3a	0.448482	0.23527	0.495002	0.0366*	
H3b	0.337177	0.276138	0.391924	0.0366*	
H4a	0.165088	0.184457	0.231378	0.0317*	
H4b	0.170457	0.089787	0.246222	0.0317*	
H5b	−0.043281	0.251622	0.273587	0.0596*	0.5
H5ax	−0.045388	0.258903	0.306961	0.0596*	0.5
H5bx	−0.112446	0.234851	0.357854	0.0596*	0.5
H6a	0.012618	0.295738	0.481032	0.0686*	0.5
H6b	0.07684	0.321573	0.418284	0.0686*	0.5
H6ax	0.014906	0.283059	0.523445	0.0686*	0.5
H6bx	0.100532	0.3335	0.433464	0.0686*	0.5
H7a	0.30989	0.165774	0.643444	0.0435*	
H7b	0.126368	0.173803	0.618669	0.0435*	
H8a	0.571696	0.15691	0.375121	0.0373*	
H8b	0.468506	0.188379	0.265919	0.0373*	
H9a	0.509646	0.0191	0.316598	0.053*	
H9b	0.399214	0.05054	0.205088	0.053*	
H10a	0.730382	0.083252	0.258209	0.0539*	
H10b	0.619781	0.107199	0.146302	0.0539*	
H11a	0.576002	−0.035328	0.099259	0.0517*	
H11b	0.685421	−0.058664	0.20906	0.0517*	
H12a	0.905875	0.002829	0.144747	0.0716*	
H12b	0.793601	0.02334	0.032196	0.0716*	
H13a	0.930204	−0.092887	0.003967	0.0839*	
H13b	0.879025	−0.137625	0.107581	0.0839*	
H13c	0.750077	−0.119484	−0.00617	0.0839*	
H5a	−0.13884	0.213923	0.366185	0.0596*	0.5

### Atomic displacement parameters (Å^2^)

**Table:**

	*U*^11^	*U*^22^	*U*^33^	*U*^12^	*U*^13^	*U*^23^
I1	0.02999 (10)	0.03331 (11)	0.03983 (12)	0.00062 (9)	0.00452 (8)	−0.01420 (10)
N1	0.0220 (11)	0.0227 (11)	0.0273 (12)	−0.0016 (10)	0.0034 (9)	0.0020 (10)
N2	0.0321 (12)	0.0270 (12)	0.0271 (14)	−0.0009 (10)	0.0056 (11)	0.0047 (10)
N3	0.0240 (11)	0.0380 (14)	0.0308 (14)	−0.0042 (11)	0.0024 (10)	0.0098 (12)
N4	0.0467 (15)	0.0317 (14)	0.0413 (17)	−0.0010 (13)	0.0168 (13)	−0.0035 (12)
C1	0.0270 (14)	0.0235 (14)	0.0285 (16)	0.0001 (11)	0.0012 (12)	0.0065 (12)
C2	0.0273 (14)	0.0336 (16)	0.0294 (16)	−0.0070 (13)	0.0045 (12)	0.0046 (13)
C3	0.0344 (15)	0.0219 (14)	0.0328 (17)	−0.0062 (12)	0.0022 (14)	0.0006 (12)
C4	0.0233 (12)	0.0310 (15)	0.0217 (14)	−0.0036 (12)	−0.0022 (11)	0.0059 (13)
C7	0.0433 (18)	0.0391 (17)	0.0265 (17)	−0.0058 (14)	0.0083 (14)	−0.0017 (13)
C8	0.0247 (13)	0.0323 (15)	0.0372 (17)	−0.0053 (13)	0.0087 (12)	0.0051 (14)
C9	0.0467 (19)	0.0394 (18)	0.052 (2)	−0.0038 (15)	0.0230 (17)	−0.0022 (16)
C10	0.0341 (16)	0.0442 (19)	0.058 (2)	−0.0009 (16)	0.0135 (16)	0.0005 (17)
C11	0.0455 (19)	0.0450 (19)	0.041 (2)	0.0046 (15)	0.0145 (17)	0.0044 (16)
C12	0.042 (2)	0.059 (2)	0.083 (3)	−0.0058 (18)	0.026 (2)	−0.012 (2)
C13	0.050 (2)	0.073 (3)	0.048 (2)	0.018 (2)	0.0190 (19)	0.000 (2)

### Geometric parameters (Å, °)

**Table:**

N1—C1	1.529 (4)	C5x—H5ax	0.96
N1—C3	1.539 (3)	C5x—H5bx	0.96
N1—C4	1.541 (3)	C6—H6a	0.96
N1—C8	1.502 (4)	C6—H6b	0.96
N2—C1	1.430 (4)	C6x—H6ax	0.96
N2—C2	1.476 (3)	C6x—H6bx	0.96
N2—C7	1.461 (4)	C7—H7a	0.96
N3—C2	1.458 (4)	C7—H7b	0.96
N3—C4	1.409 (4)	C8—C9	1.503 (4)
N3—C5	1.411 (6)	C8—H8a	0.96
N3—C5x	1.441 (6)	C8—H8b	0.96
N4—C3	1.408 (4)	C9—C10	1.490 (5)
N4—C6	1.438 (7)	C9—H9a	0.96
N4—C6x	1.412 (7)	C9—H9b	0.96
N4—C7	1.452 (4)	C10—C11	1.500 (5)
C1—H1a	0.96	C10—H10a	0.96
C1—H1b	0.96	C10—H10b	0.96
C2—H2a	0.96	C11—C12	1.499 (5)
C2—H2b	0.96	C11—H11a	0.96
C3—H3a	0.96	C11—H11b	0.96
C3—H3b	0.96	C12—C13	1.491 (6)
C4—H4a	0.96	C12—H12a	0.96
C4—H4b	0.96	C12—H12b	0.96
C5—C6	1.439 (10)	C13—H13a	0.96
C5—H5b	0.96	C13—H13b	0.96
C5—H5a	0.96	C13—H13c	0.96
C5x—C6x	1.435 (10)		
			
C1—N1—C3	106.5 (2)	N4—C6—C5	132.1 (6)
C1—N1—C4	106.25 (19)	N4—C6—H6a	109.4707
C1—N1—C8	112.7 (2)	N4—C6—H6b	109.4714
C3—N1—C4	111.58 (19)	C5—C6—H6a	109.4715
C3—N1—C8	108.7 (2)	C5—C6—H6b	109.4717
C4—N1—C8	111.0 (2)	H6a—C6—H6b	69.7221
C1—N2—C2	109.3 (2)	N4—C6x—C5x	101.0 (6)
C1—N2—C7	109.5 (2)	N4—C6x—H6ax	109.4706
C2—N2—C7	112.7 (2)	N4—C6x—H6bx	109.4714
C2—N3—C4	111.8 (2)	C5x—C6x—H6ax	109.4706
C2—N3—C5	121.2 (4)	C5x—C6x—H6bx	109.4716
C2—N3—C5x	113.1 (4)	H6ax—C6x—H6bx	116.7784
C4—N3—C5	118.6 (4)	N2—C7—N4	113.3 (3)
C4—N3—C5x	113.9 (4)	N2—C7—H7a	109.4714
C3—N4—C6	111.0 (4)	N2—C7—H7b	109.472
C3—N4—C6x	121.9 (4)	N4—C7—H7a	109.4709
C3—N4—C7	112.4 (2)	N4—C7—H7b	109.4705
C6—N4—C7	115.0 (4)	H7a—C7—H7b	105.3282
C6x—N4—C7	116.7 (4)	N1—C8—C9	116.5 (2)
N1—C1—N2	109.8 (2)	N1—C8—H8a	109.4716
N1—C1—H1a	109.4711	N1—C8—H8b	109.4713
N1—C1—H1b	109.4708	C9—C8—H8a	109.4715
N2—C1—H1a	109.4712	C9—C8—H8b	109.4708
N2—C1—H1b	109.4718	H8a—C8—H8b	101.3702
H1a—C1—H1b	109.0907	C8—C9—C10	113.0 (3)
N2—C2—N3	112.6 (2)	C8—C9—H9a	109.4702
N2—C2—H2a	109.4708	C8—C9—H9b	109.4711
N2—C2—H2b	109.472	C10—C9—H9a	109.472
N3—C2—H2a	109.4708	C10—C9—H9b	109.4714
N3—C2—H2b	109.4711	H9a—C9—H9b	105.7374
H2a—C2—H2b	106.0941	C9—C10—C11	115.5 (3)
N1—C3—N4	113.7 (2)	C9—C10—H10a	109.4709
N1—C3—H3a	109.4712	C9—C10—H10b	109.471
N1—C3—H3b	109.4711	C11—C10—H10a	109.4711
N4—C3—H3a	109.4713	C11—C10—H10b	109.4722
N4—C3—H3b	109.4714	H10a—C10—H10b	102.6643
H3a—C3—H3b	104.9048	C10—C11—C12	117.3 (3)
N1—C4—N3	112.3 (2)	C10—C11—H11a	109.4708
N1—C4—H4a	109.4714	C10—C11—H11b	109.4718
N1—C4—H4b	109.4716	C12—C11—H11a	109.4709
N3—C4—H4a	109.471	C12—C11—H11b	109.4715
N3—C4—H4b	109.4715	H11a—C11—H11b	100.2535
H4a—C4—H4b	106.513	C11—C12—C13	115.6 (3)
N3—C5—C6	103.0 (5)	C11—C12—H12a	109.4712
N3—C5—H5b	109.4716	C11—C12—H12b	109.4718
N3—C5—H5a	109.472	C13—C12—H12a	109.4714
C6—C5—H5b	109.4704	C13—C12—H12b	109.4707
C6—C5—H5a	109.4707	H12a—C12—H12b	102.5339
H5b—C5—H5a	115.2131	C12—C13—H13a	109.4716
N3—C5x—C6x	134.1 (6)	C12—C13—H13b	109.471
N3—C5x—H5ax	109.4707	C12—C13—H13c	109.4712
N3—C5x—H5bx	109.471	H13a—C13—H13b	109.4712
C6x—C5x—H5ax	109.4709	H13a—C13—H13c	109.4704
C6x—C5x—H5bx	109.4719	H13b—C13—H13c	109.4719
H5ax—C5x—H5bx	62.4606		
			
?—?—?—?	?		

### Hydrogen-bond geometry (Å, °)

**Table:**

*D*—H···*A*	*D*—H	H···*A*	*D*···*A*	*D*—H···*A*
C1—H1a···I1^i^	0.96	2.98	3.913 (3)	164
C3—H3b···I1^ii^	0.96	3.04	3.925 (2)	154

Symmetry codes: (i) −*x*+1, −*y*, −*z*+1; (ii) *x*−1/2, −*y*+1/2, *z*−1/2.
